# A checklist of helminth parasites of Elasmobranchii in Mexico

**DOI:** 10.3897/zookeys.563.6067

**Published:** 2016-02-15

**Authors:** Aldo Iván Merlo-Serna, Luis García-Prieto

**Affiliations:** 1 Laboratorio de Helmintología, Instituto de Biología, Universidad Nacional Autónoma de México, Ap. Postal 70-153, C.P. 04510, México D.F., México

**Keywords:** Platyhelminthes, Nematoda, Hirudinea, Sharks, Rays, Richness, Selachii, Batoidea

## Abstract

A comprehensive and updated summary of the literature and unpublished records contained in scientific collections on the helminth parasites of the elasmobranchs from Mexico is herein presented for the first time. At present, the helminth fauna associated with Elasmobranchii recorded in Mexico is composed of 132 (110 named species and 22 not assigned to species), which belong to 70 genera included in 27 families (plus 4 *incertae sedis* families of cestodes). These data represent 7.2% of the worldwide species richness. Platyhelminthes is the most widely represented, with 128 taxa: 94 of cestodes, 22 of monogeneans and 12 of trematodes; Nematoda and Annelida: Hirudinea are represented by only 2 taxa each. These records come from 54 localities, pertaining to 15 states; Baja California Sur (17 sampled localities) and Baja California (10), are the states with the highest species richness: 72 and 54 species, respectively. Up to now, 48 elasmobranch species have been recorded as hosts of helminths in Mexico; so, approximately 82% of sharks and 67% of rays distributed in Mexican waters lack helminthological studies. The present list provides the host, distribution (with geographical coordinates), site of infection, accession number in scientific collections, and references for the parasites. A host-parasite list is also provided.

## Introduction

According to Eschmeyer and Fong (2015), 1338 species of elasmobranchs have been described worldwide (768 rays and 570 sharks). However, Naylor et al. (2012), based on the fact that since 2005 more than 130 new species have been described, considered that more species remain to be discovered. According to these authors, this increase is a result of reassessment of geographic variation; some of the increase represents recognition of subtle morphological variants among congeneric forms that nevertheless exhibit substantial molecular sequence divergence (cryptic species). In Mexico, this group is represented by 204 known species (95 rays and 109 sharks) (Del Moral-Flores and Pérez-Ponce de León 2013) (Table 1); this richness constitutes 15% of the living species in the world. Nonetheless, most of the species recorded in Mexican waters also have been found in international waters, and many of them are cosmopolitan (Espinoza-Pérez et al. 2004).

**Table 1.** Species of Elasmobranchii reported from Mexico and richness of associated helminths (modified from Del Moral-Flores and Pérez-Ponce de León 2013).

Subclass	Order	Family	Genera	Species	Sampled species	Helminth recorded
**Selachii**	Hexanchiformes	Chlamydoselachidae	1	1	0	0
		Hexanchidae	3	4	1	1
		Echinorhinidae	1	1	0	0
		Squalidae	2	4	0	0
		Centrophoridae	1	4	0	0
	Squaliformes	Etmopteridae	2	8	0	0
		Somniosidae	3	3	0	0
		Oxynotidae	1	1	0	0
		Dalatiidae	4	5	0	0
	Squatiniformes	Squatinidae	1	4	1	2
	Heterodontiformes	Heterodontidae	1	2	2	7
		Ginglymostomatidae	1	1	1	3
	Orectolobiformes	Rhicodontidae	1	1	0	0
		Odontaspididae	2	3	0	0
		Pseudocarcharhiidae	1	1	0	0
		Megachasmidae	1	1	0	0
	Lamniformes	Alopidae	1	4	2	6
		Cetorhinidae	1	1	0	0
		Lamnidae	3	4	0	0
		Scyliorhinidae	6	15	0	0
		Triakidae	3	11	5	9
	Carcharhiniformes	Carcharhinidae	7	25	5	19
		Sphyrnidae	1	6	3	5
TOTALS^*^	7	23	48	109	20	52
**Batoidea**	Torpediniformes	Torpedinidae	1	2	0	0
		Narcinidae	2	4	2	5
	Pristiformes	Pristidae	1	3	0	0
		Rhinobatidae	2	10	5	10
	Rhinobatiformes	Platyrhinidae	1	1	0	0
		Arhynchobatidae	2	4	0	0
	Rajiformes	Rajidae	9	29	2	2
		Anacanthobatidae	2	4	1	1
		Urotrygonidae	2	10	5	27
		Dasyatidae	3	9	5	26
		Gymnuridae	1	4	0	0
	Myliobatiformes	Myliobatidae	3	8	8	38
		Rhinopteridae	1	2	0	0
		Mobulidae	2	6	0	0
TOTALS^*^	5	14	32	95	28	108

* The totals in the table are greater than in the text because some species infect 2 or more host species (sharks and/or rays).

Elasmobranchs (sharks, skates and rays) are host to a great variety of parasites in nature, particularly helminths. Up to now, more than 1500 helminth species have been recorded in association with these hosts worldwide; cestodes represent the most diverse group, with approximately 1133 species, followed by monogeneans with 226, nematodes with 83, digeneans with 50-60, leeches with 23, and aspidogastreans with 2 (Caira et al. 2012). In addition, 4 species of acanthocephalans have been found only in elasmobranchs (Weaver and Smales 2014). In Mexico, the first record of a helminth parasitizing an elasmobranch was made by Caballero y Caballero (1945), who described the digenean *Staphylorchis pacifica* (=*Petalodistomum pacificum*) from the body cavity of an undetermined shark in the Pacific slope of this country. Since then, a great amount of information has been generated, most of it in the last 2 decades, particularly in the Gulf of California. The main goal of this checklist is to compile and discuss all these data and to establish some patterns of richness, geographical distribution and host spectrum.

## Methods

This checklist contains information updated until December, 2015, and comes from two different sources: 1) retrospective bibliographical search, using different databases such as CAB Abstracts, Biological Abstracts, and Zoological Record; 2) Search in databases of national [Colección Nacional de Helmintos (CNHE), Instituto de Biología, UNAM, Mexico City, Mexico] and international [Harold W. Manter Laboratory of Parasitology (HWML), University of Nebraska-Lincoln, USA; National Museum of Natural History (USNM), Smithsonian Institution, Washington, D.C., USA, formerly United States National Parasite Collection (USNPC), Beltsville, Maryland, USA] parasite collections.

The checklist is divided into two sections; the first includes a parasite-host list, presented in phylogenetic order, starting with the phylum Platyhelminthes (Trematoda, Monogenoidea and Cestoda), and followed by the phyla Nematoda and Annelida (Hirudinea). Each phylum contains families, genera, and species in alphabetical order. The nomenclature and classification for each metazoan group is based on the following references: Trematoda (Gibson et al. 2002; Jones et al. 2005; Bray et al. 2008), Monogenoidea (Boeger and Kritsky 1993), Cestoda (Caira et al. 2014b), Nematoda (Anderson et al. 1974–1983; Gibbons 2010), and Hirudinea (Sawyer 1986; Davies 1991). The information for each helminth species includes species name, authority, and site of infection. We use “NA” when some data are not available in the original source. Next, we present species distributions, referring to states of the Mexican Republic (in caps) where the record was made as well as the specific locality name, followed by the species of host and the bibliographic references related to records. For specimens deposited in a collection, acronyms are as follows:

BMNH
 The British Museum (Natural History) Collection at the Natural History Museum, London, UK 


CNHE
 Colección Nacional de Helmintos, Instituto de Biología, UNAM, Mexico City, México 


CPMHN-UABCS
 Colección Parasitológica del Museo de Historia Natural de la Universidad Autónoma de Baja California Sur, La Paz, Baja California Sur, Mexico 


ECOPA
El Colegio de la Frontera Sur, Chetumal, Quintana Roo, Mexico 


HWML
 Harold W. Manter Laboratory of Parasitology, University of Nebraska-Lincoln, Nebraska, United States 


IPCAS
 Institute of Parasitology, Academy of Sciences of the Czech Republic, Česke Budějovice, Czech Republic 


LRP
 Lawrence R. Penner Collection, Department of Ecology and Evolutionary Biology, University of Connecticut, Storrs CT, USA 


MNHG-INV or PLAT Museum of Natural History, Geneva, Switzerland


SBMNH
Santa Barbara Museum of Natural History, Santa Barbara, California, United States 


TINRO
 Pacific Fisheries Research Center, Vladivostok, Russian Federation 


UCLA
 Helminthological Collection, Zoology Department, University of California at Los Angeles 


USNPC
 Accession numbers used in this work correspond to those given by United States National Parasite Collection, Beltsville, Maryland, USA, which was recently transferred to the National Museum of Natural History (USMN), Smithsonian Institution, Washington, D.C., USA 


The name of the type locality (TL), type host (TH), and original reference (OR) of the new species described from elasmobranchs recorded in Mexico are indicated with abbreviations of these words in superscript.

The host-parasite list is ordered alphabetically by families of elasmobranchs; each family includes the scientific name of the host and the authority name. Then, the scientific names of the species of helminths are listed in alphabetical order, indicating in parentheses the parasite group to which they belong. The scientific names of elasmobranchs were updated following Froese and Pauly (2014); higher levels of classification follow Del Moral-Flores and Pérez-Ponce de León (2013).

## Results

**Figure 1.**
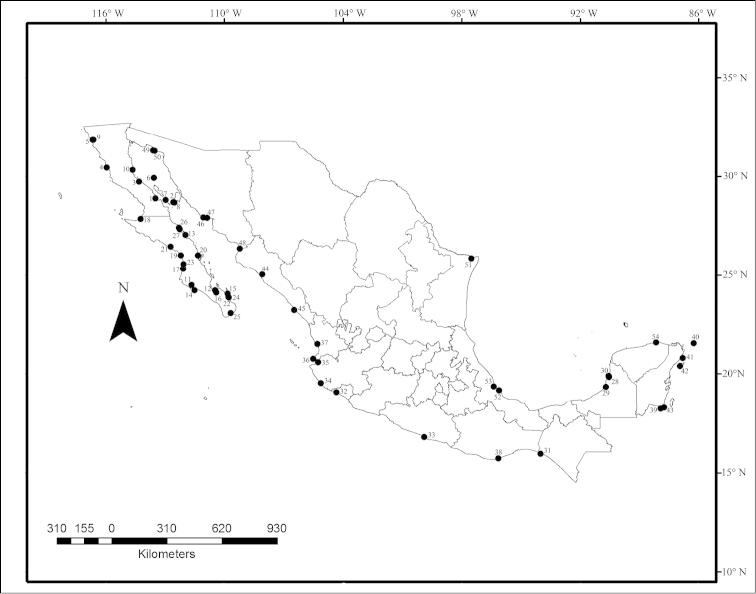
Map of Mexico showing the localities that have been sampled for elasmobranchs as hosts of helminth species.

To date, 48 species of elasmobranchs (20 sharks and 28 rays) have been recorded as host of 132 taxa of helminths (110 named species and 22 not assigned to species); these parasite species belong to 70 genera included in 27 families (plus 4 families of cestodes that are *incertae sedis*). Platyhelminthes is represented by 128 taxa: 94 taxa of cestodes, 22 taxa of monogeneans and 12 taxa of trematodes; for both Nematoda and Annelida (Hirudinea) only 2 species have been recorded. The 54 sampled sites for helminths are located in 15 states; Baja California Sur (17 localities) and Baja California (10), are the states with the highest species richness (72 and 54, respectively) (Fig. 1). Up to now, no helminths parasitizing elasmobranchs from Mexican waters have been reported from the states of Michoacán and Tabasco; for Chiapas, Colima, Tamaulipas and Yucatán, only one record each has been reported. Below, we present the checklist of helminth parasites recorded in elasmobranch species caught in Mexico, which summarizes the current knowledge on this group in the country.

### Parasite-host list

**Trematoda Rudolphi, 1808**

**Acanthocolpidae Lühe, 1906**

***Pleorchis magniporus* Arai, 1962**

**Site of infection.** Intestine.

**Locality.** BAJA CALIFORNIA SUR: Bahía Magdalena^TL^: *Urolophus maculatus*^TH^ (see Arai 1962^OR^).

**Specimens in collections.** UCLA.

**Azygiidae Lühe, 1909**

***Otodistomum veliporum* (Creplin, 1837) Stafford, 1904**

**Site of infection.** Body cavity, stomach.

**Locality.** BAJA CALIFORNIA SUR: Santa Rosalía: *Heterodontus francisci*, *Heterodontus mexicanus*, *Mustelus henlei*, *Squatina californica* (see Curran and Overstreet 2000).

**Specimens in collections.** CNHE (3852).

**Bucephalidae Poche, 1907**

***Prosorhynchus truncatus* Verma, 1936**

**Site of infection.** Intestine.

**Locality.** BAJA CALIFORNIA SUR: El Comitán: *Dasyatis brevis* (see Villarreal-Lizárraga 1995).

**Specimens in collections.** CPMHN-UABCS (20).

**Gorgoderidae Looss, 1899**

***Anaporrhutum euzeti* Curran, Blend & Overstreet, 2003**

**Site of infection.** Pericardial and body cavities.

**Locality.** BAJA CALIFORNIA: Bahía de Los Ángeles: *Dasyatis brevis* (see Curran et al. 2003). BAJA CALIFORNIA SUR: Loreto^TL^: *Myliobatis longirostris*^TH^ (see Curran et al. 2003^OR^). NA: Golfo de California: *Dasyatis longa*, *Diplobatis ommata*, *Mobula munkiana*, *Myliobatis californica*, *Narcine entemedor*, *Rhinobatos productus*, *Urolophus halleri*, *Urolophus maculatus*, *Zapteryx exasperata* (see Curran et al. 2013)

**Specimens in collections.** CNHE (4499); HWML (16702); SBMNH (345780).

***Nagmia cisloi* Curran, Blend & Overstreet, 2009**

**Site of infection.** Body cavity.

**Locality.** BAJA CALIFORNIA SUR: Bahía de La Paz^TL^: *Mobula thurstoni*^TH^ (see Curran et al. 2009^OR^).

**Specimens in collections.** CNHE (6198).

***Nagmia rodmani* Curran, Blend & Overstreet, 2009**

**Site of infection.** Body cavity.

**Locality.** BAJA CALIFORNIA SUR: Loreto ^TL^: *Narcine entemedor* ^TH^ (see Curran et al. 2009 ^OR^).

**Specimens in collections.** CNHE (6199); HWML (48889); SBMNH (423115).

***Probolitrema richiardii* (López, 1888) Looss, 1902**

**Site of infection.** Body cavity.

**Locality.** BAJA CALIFORNIA: Isla San Esteban: *Urobatis* sp. (see Curran et al. 2009). BAJA CALIFORNIA SUR: Bahía de Santa Inés: *Dasyatis brevis*, *Mustelus lunulatus*, *Urolophus maculatus* (see Markell 1956). NA: Golfo de California: *Dasyatis brevis*, *Dasyatis longa*, *Myliobatis californica*, *Myliobatis longirostris*, *Rhinobatos leucorhynchus* (see Curran et al. 2009)

**Specimens in collections.** CNHE (6200); HWML (48890); SBMNH (423116); USNPC (49354).

**Notes.** The specimens of Bahía de Santa Inés were identified as *Probolitrema mexicana*, but this species is a synonym of *Probolitrema richiardi* according to Curran et al. (2009).

***Staphylorchis pacifica* (Caballero y Caballero, 1945) Campbell, 2008**

**Site of infection.** Body cavity.

**Locality.** COLIMA: Manzanillo ^TL^: “Tiburón no determinado^TH^” (see Caballero y Caballero 1945^OR^). JALISCO: Puerto Vallarta: “Elasmobranchii” (CNHE); NAYARIT: Punta Mita: “Tiburón no determinado” (see Bravo-Hollis 1954); San Blás: *Carcharhunus limbatus* (see Lamothe-Argumedo 1969). SINALOA: Mazatlán: *Galeorhinus galeus* (see Winter 1959).

**Specimens in collections.** CNHE (921, 1069, 1111, 1585, 3246).

**Notes.** The original description of this species was made under the name *Petalodistomum pacificum* (Caballero y Caballero 1945); later, this species was transferred to *Nagmia* by Markell (1953). This act was accepted by Sogandares-Bernal (1959) and Curran et al. (2009) but rejected by Caballero y Caballero et al. (1956) and Winter (1959). Lamothe-Argumedo (1969) erected *Winteria* to accommodate this species, but this genus was considered a synonym of *Nagmia* (Curran et al. 2009). Pigulevski (1953) divided the genus *Petalodistomum* in 2 subgenera, including the species of *Petalodistomum* described by Caballero y Caballero (1945) in Petalodistomum (Petalodistomum). Currently, this trematode species is accepted as *Staphylorchis pacifica* (see Campbell 2008).

**Opecoelidae Ozaki, 1925**

***Helicometrina nimia* Linton, 1910**

**Site of infection.** Stomach.

**Locality.** BAJA CALIFORNIA SUR: Las Barrancas: *Prionace glauca* (see Méndez 2005).

**No specimens in collections.**

**Ptychogonimidae Dollfus, 1937**

***Ptychogonimus megastomum* (Rudolphi, 1819) Lühe, 1900**

**Site of infection.** Stomach.

**Locality.** BAJA CALIFORNIA: Bahía de Los Ángeles: *Mustelus californicus* (see Curran and Overstreet 2000); Puertecitos: *Mustelus lunulatus* (see Curran and Overstreet 2000).

**Specimens in collections.** CNHE (3853-4).

**Syncoeliidae Loos, 1899**

***Paronatrema vaginicola* Dollfus, 1937**

**Site of infection.** Buccal cavity, cloaca, gills.

**Locality.** BAJA CALIFORNIA SUR: Boca de Álamo: *Alopias pelagicus*, *Prionace glauca* (see Curran and Overstreet 2000); Puntarena, San Isidro: *Prionace glauca* (see Curran and Overstreet 2000). SINALOA: Bahía Santa María: *Alopias pelagicus* (see Curran and Overstreet 2000).

**Specimens in collections.** CNHE (3855); HWML (15263, 15265).

***Syncoelium vermilionensis* Curran & Overstreet, 2000**

**Site of infection.** Gills.

**Locality.** BAJA CALIFORNIA SUR: Puntarena: *Mobula japanica* (see Curran and Overstreet 2000); Santa María^TL^: *Mobula thurstoni* ^TH^ (see Curran and Overstreet 2000^OR^).

**Specimens in collections.** CNHE (3850); HWML (15261).

**Monogenoidea Bychowsky, 1937**

**Capsalidae Baird, 1853**

***Benedeniella posterocolpa* (Hargis, 1955) Yamaguti, 1963**

**Site of infection.** Skin.

**Locality.** CAMPECHE: Estuario Champotón: *Rhinoptera bonasus* (see Pulido-Flores and Monks 2005).

**Specimens in collections.** CNHE (4370).

***Listrocephalos guberleti* (Caballero y Caballero & Bravo-Hollis, 1962) Bullard, Payne & Braswell, 2004**

**Site of infection.** Gills, skin.

**Locality.** BAJA CALIFORNIA: Bahía de Los Ángeles: *Urolophus halleri* (see Bullard et al. 2004); Isla San Esteban: *Urobatis concentricus*, *Urolophus maculatus*, *Urobatis* sp. (see Bullard et al. 2004). SONORA: Bahía de Guaymas^TL^: *Urolophus halleri* ^TH^ (see Caballero y Caballero and Bravo-Hollis 1962^OR^).

**Specimens in collections.** CNHE (34-5); USNPC (94826-8).

**Notes.** This species was described as *Entobdella guberleti* (Caballero y Caballero and Bravo-Hollis 1962) and transferred to *Listrocephalos* by Bullard et al. (2004).

***Listrocephalos kearni* Bullard, Payne & Braswell, 2004**

**Site of infection.** Skin.

**Locality.** BAJA CALIFORNIA: Bahía de Los Ángeles^TL^: *Dasyatis brevis* ^TH^ (see Bullard et al. 2004 ^OR^). BAJA CALIFORNIA SUR: Santa Rosalía: *Dasyatis brevis* (see Bullard et al. 2004).

**Specimens in collections.** CNHE (5021-2); USNPC (94829-34).

***Listrocephalos whittingtoni* Bullard, Payne & Braswell, 2004**

**Site of infection.** Skin.

**Locality.** BAJA CALIFORNIA: Bahía de Los Ángeles^TL^: *Dasyatis longa* ^TH^ (see Bullard et al. 2004^OR^). BAJA CALIFORNIA SUR: Bahía de La Paz: *Dasyatis longa* (see Bullard et al. 2004).

**Specimens in collections.** CNHE (5023-4); USNPC (94835-9).

**Hexabothriidae Price, 1942**

***Dasyonchocotyle dasyatis* (Yamaguti, 1968) Boeger & Kritsky, 1989**

**Site of infection.** Gills.

**Locality.** SINALOA: Mazatlán: *Dasyatis longa* (see Escorcia-Ignacio et al. 2015).

**Specimens in collections.** CNHE (9361).

**Loimoidae Price, 1936**

***Loimos winteri* Caballero y Caballero & Bravo-Hollis, 1961**

**Site of infection.** Gills.

**Locality.** SONORA: Bahía de Guaymas^TL^: *Carcharhinus brachyurus*^TH^ (see Caballero y Caballero and Bravo-Hollis 1961^OR^).

**Specimens in collections.** CNHE (86-7).

***Loimosina parawilsoni* Bravo-Hollis, 1970**

**Site of infection.** Gills.

**Locality.** SINALOA: Mazatlán^TL^: *Sphyrna lewini* ^TH^ (see Bravo-Hollis 1970^OR^).

**Specimens in collections.** CNHE (153-4).

**Monocotylidae Taschenberg, 1879**

***Anoplocotyloides papillatus* (Doran, 1953) Young, 1967**

**Site of infection.** Gills.

**Locality.** SINALOA: Mazatlán: *Rhinobatos glaucostigma* (see Bravo-Hollis 1969).

**Specimens in collection.** CNHE (178).

**Notes.** Based on the morphology of the posterior hooks of the haptor, Neifar et al. (2002) considered that this material is composed of 2 different monocotylideans.

***Calicotyle californiensis* Bullard & Overstreet, 2000**

**Site of infection.** Body cavity.

**Locality.** BAJA CALIFORNIA: Bahía de Los Ángeles^TL^: *Mustelus californicus*^TH^ (see Bullard and Overstreet 2000^OR^).

**Specimens in collections.** CNHE (3907).

***Calicotyle kroyeri* Diesing, 1850**

**Site of infection.** Cloaca, rectum.

**Locality.** CAMPECHE: Bancos de Campeche: *Anacanthobatis folirostris*, *Dipturus olseni* (see Chisholm et al. 1997).

**No specimens in collections.**

***Calicotyle urobati* Bullard & Overstreet, 2000**

**Site of infection.** Cloaca, rectum.

**Locality.** BAJA CALIFORNIA: Bahía de Los Ángeles^TL^: *Urolophus halleri* ^TH^, *Urolophus maculatus* (see Bullard and Overstreet 2000^OR^); Bahía de San Francisquito: *Urolophus halleri* (see Bullard and Overstreet 2000); Puertecitos: *Urolophus maculatus* (see Bullard and Overstreet 2000). BAJA CALIFORNIA SUR: Santa Rosalía: *Urolophus halleri*, *Urolophus maculatus* (see Bullard and Overstreet 2000).

**Specimens in collections.** CNHE (3908-9); HWML (15365-6); USNPC (89777-8).

**Dasybatotreminae gen. sp.**

**Site of infection.** Gills.

**Locality.** GUERRERO: Acapulco: *Rhinoptera steindachneri* (see Carbajal-Violante 2012).

**Specimens in collections.** CNHE (8287-8).

***Decacotyle floridana* (Pratt, 1910) Chisholm & Whittington, 1998**

**Site of infection.** Gills.

**Locality.** CAMPECHE: Ciudad del Carmen: *Aetobatus narinari* (CNHE); Estuario Champotón: *Aetobatus narinari* (see Pulido-Flores and Monks 2005). QUINTANA ROO: Holbox: *Aetobatus narinari* (see Pulido-Flores and Monks 2005).

**Specimens in collections.** CNHE (327, 4368).

**Notes.** Specimens from Ciudad del Carmen were identified as *Heterocotyle aetobatis* Hargis, but this species was considered a synonym of *Decacotyle floridana* by Chisholm and Whittington (1998).

***Denarycotyle gardneri* Pulido-Flores, Monks & Violante-González, 2015**

**Site of infection.** Gills.

**Locality.** GUERRERO: Acapulco^TL^: *Rhinoptera steindachneri*^TH^ (Pulido-Flores et al. 2015^OR^).

**Specimens in collections.** CNHE (9558-9); HWML (75364-7).

***Dendromonocotyle cortesi* Bravo-Hollis, 1969**

**Site of infection.** Skin.

**Locality.** BAJA CALIFORNIA: Bahía de Los Ángeles^TL^, Isla Rasa: “Mantarraya gris^TH^” (see Bravo-Hollis 1969^OR^) .

**Specimens in collections.** CNHE (149-50).

**Notes.** Valid species according to Chisholm et al. (2004).

***Dendromonocotyle octodiscus* Hargis, 1955**

**Site of infection.** Skin.

**Locality.** GOLFO DE MEXICO (Mexico): *Dasyatis americana*, *Urobatis jamaicensis* (see Fehlauer-Ale and Littlewood 2011). QUINTANA ROO: Blanquizal, Holbox: *Dasyatis americana* (see Pulido-Flores and Monks 2005); El Paso de los Cedros (Cozumel), Ixmapuit (Isla Contoy), Xcalak: *Urobatis jamaicensis* (see Pulido-Flores and Monks 2005). YUCATÁN: Ría Lagartos: *Urobatis jamaicensis* (see Pulido-Flores and Monks 2005).

**Specimens in collections.** CNHE (4362-3, 4366-7); ECOPA (001); USNPC (90353).

**Notes.** Valid species accordig to Chisholm et al. (2004).

***Euzetia lamothei* Pulido-Flores & Monks, 2008**

**Site of infection.** Gills.

**Locality.** CAMPECHE: Ciudad del Carmen^TL^: *Rhinoptera bonasus*^TH^ (see Pulido-Flores and Monks 2008^OR^). QUINTANA ROO: Isla Contoy: *Rhinoptera bonasus* (see Pulido-Flores and Monks 2008).

**Specimens in collections.** CNHE (6067-8); HWML (48817); CHE (P00056).

***Heterocotyle* sp.**

**Site of infection.** Gills.

**Locality.** GUERRERO: Acapulco: *Rhinoptera steindachneri* (see Carbajal-Violante 2012).

**No specimens in collections.**

**Monocotylidae gen. sp.**

**Site of infection.** Gills.

**Locality.** BAJA CALIFORNIA SUR: Bahía Almejas: *Rhinoptera steindachneri* (see Gómez del Prado-Rosas and Euzet 1997).

**No specimens in collections.**

**Notes.** This material was recorded as *Quadritestis almehensis* n. gen., n. sp., but its description was not published, so that name is a *nomen nudum*.

***Spinuris lophosoma* Doran, 1953**

**Site of infection.** Gills.

**Locality.** BAJA CALIFORNIA SUR: Bahía Almejas: *Rhinobatos productus* (see Gómez del Prado-Rosas and Euzet 1999).

**No specimens in collections.**

***Spinuris mexicana* Bravo-Hollis, 1969**

**Site of infection.** Gills.

**Locality.** SINALOA: Mazatlán^TL^: *Rhinobatos glaucostigma*^TH^ (see Bravo-Hollis 1969^OR^).

**Specimens in collections.** CNHE (151-2).

***Spinuris zapterygis* Gómez del Prado-Rosas & Euzet, 1999**

**Site of infection.** Gills.

**Locality.** BAJA CALIFORNIA SUR: Bahía Almejas^TL^: *Zapteryx exasperata*^TH^ (see Gómez del Prado-Rosas and Euzet 1999 ^OR^).

**Specimens in collections.** BM(NH) (1997.1.28.1); CNHE (2975-6); CPMHN-UABCS (54); MNHN (547HF Tk80); USNPC (87037).

**Cestoda Rudolphi, 1808**

**Anthocephaliidae Ruhnke, Caira & Cox, 2015**

***Anthocephalum currani* Ruhnke & Seaman, 2009**

**Site of infection.** Spiral valve.

**Locality.** BAJA CALIFORNIA: Bahía de Los Ángeles, Puertecitos: *Dasyatis brevis* (see Ruhnke and Seaman 2009); Bahía de Los Ángeles: *Dasyatis dipterura* (see Ruhnke et al. 2015). BAJA CALIFORNIA SUR: Puntarena^TL^: *Dasyatis brevis*^TH^ (see Ruhnke and Seaman 2009^OR^).

**Specimens in collections.** CNHE (6234-5); USNPC (100993).

**Notes.** This species was identified as *Anthocephalum* n. sp. 2. in Olson et al.’s (1999) phylogenetic analysis.

***Anthocephalum duszynskii* Ruhnke, 1994**

**Site of infection.** Spiral valve.

**Locality.** SONORA: Puerto Peñasco (Bahía Cholla)^TL^: *Urolophus halleri*^TH^ (see Ruhnke 1994 ^OR^).

**Specimens in collections.** HWML (37095); USNPC (83437).

***Anthocephalum lukei* Ruhnke & Seaman, 2009**

**Site of infection.** Spiral valve.

**Locality.** BAJA CALIFORNIA: Bahía de Los Ángeles, Puertecitos^TL^: *Dasyatis longa*^TH^ (see Ruhnke and Seaman 2009^OR^). BAJA CALIFORNIA SUR: Bahía de La Paz: *Dasyatis longa* (see Ruhnke and Seaman 2009).

**Specimens in collections.** CNHE (6232-3); USNPC (100995).

**Notes.** This species was identified as *Anthocephalum* n. sp. 1. in the Olson et al.’s (1999) phylogenetic analysis.

***Anthocephalum michaeli* Ruhnke & Seaman, 2009**

**Site of infection.** Spiral valve.

**Locality.** BAJA CALIFORNIA: Bahía de Los Ángeles: *Dasyatis longa* (see Ruhnke and Seaman 2009); Isla San Esteban: *Urolophus maculatus* (see Caira et al. 2001). BAJA CALIFORNIA SUR: Bahía de La Paz, Loreto^TL^, Puntarena, San José del Cabo: *Dasyatis longa*^TH^ (see Ruhnke and Seaman 2009^OR^).

**Specimens in collections.** CNHE (6230-1); LRP (4232); USNPC (100998-9, 101000).

**Notes.** Specimens from Isla San Esteban, identified as *Anthocephalum duszynskii* by Caira et al. (2001), were re-identified as *Anthocephalum michaeli* by Ruhnke and Seaman (2009).

**Cathetocephalidae Dailey & Overstreet, 1973**

***Cathetocephalus resendezi* Caira, Mega & Ruhnke, 2005**

**Site of infection.** Spiral valve.

**Locality.** BAJA CALIFORNIA: Bahía de Los Ángeles^TL^: *Carcharhinus leucas*^TH^ (see Caira et al. 2005^OR^).

**Specimens in collections.** CNHE (5300).

***Cathetocephalus thatcheri* Dailey & Overstreet, 1973**

**Site of infection.** Spiral valve.

**Locality.** VERACRUZ: Playa Chachalacas: *Carcharhinus leucas* (see Méndez and Dorantes 2013).

**Specimens in collections.** CNHE (6860).

**Echeneibothriidae de Beauchamp, 1905**

***Echeneibothrium* sp.**

**Site of infection.** Spiral valve.

**Locality.** BAJA CALIFORNIA: Santa Rosalía: *Myliobatis californicus* (see Caira et al. 1999), *Raja velezi* (see Healy et al. 2009).

**Specimens in collections.** LRP (4217).

**Notes.** This material was recorded as *Discobothrium* sp., but according to Euzet (1994), this genus is a synomym of *Echeneibothrium*.

**Echinobothriidae Perrier, 1897**

***Echinobothrium fautleyae* Tyler & Caira, 1999**

**Site of infection.** Spiral valve.

**Locality.** BAJA CALIFORNIA: Bahía de Los Ángeles^TL^: *Rhinoptera steindachneri*^TH^ (see Tyler and Caira 1999 ^OR^); Puertecitos: *Myliobates californica*, *Rhinoptera steindachneri* (see Tyler and Caira 1999). BAJA CALIFORNIA SUR: Loreto: *Rhinoptera steindachneri* (see Tyler and Caira 1999); Puntarena: *Rhinoptera steindachneri* (see Tyler and Caira 1999); Santa Rosalía: *Myliobates californica*, *Rhinoptera steindachneri* (see Tyler and Caira 1999).

**Specimens in collections.** CNHE (3340-1); HWML (33910-11); USNPC (88217-19).

***Echinobothrium hoffmanorum* Tyler, 2001**

**Site of infection.** Spiral valve.

**Locality.** BAJA CALIFORNIA: Bahía de San Francisquito: *Urolophus halleri*, *Urolophus maculatus* (see Tyler 2001); Isla San Esteban^TL^: *Urolophus maculatus*^TH^ (see Tyler 2001^OR^). BAJA CALIFORNIA SUR: Puntarena: *Urobatis concentricus* (see Tyler 2001).

**Specimens in collections.** CNHE (3916-9).

***Echinobothrium mexicanum* Tyler & Caira, 1999**

**Site of infection.** Spiral valve.

**Locality.** BAJA CALIFORNIA: Bahía de Los Ángeles^TL^: *Myliobatis longirostris*^TH^, *Myliobatis californica* (see Tyler and Caira 1999^OR^); Puertecitos: *Myliobatis californica* (see Tyler and Caira 1999). BAJA CALIFORNIA SUR: Loreto: *Myliobatis longirostris*, *Myliobatis californica* (see Tyler and Caira 1999); Santa Rosalía: *Myliobatis longirostris* (see Tyler and Caira 1999).

**Specimens in collections.** CNHE (3343-5); HWML (33912-14); USNPC (88220-21).

***Echinobothrium rayallemangi* Tyler, 2001**

**Site of infection.** Spiral valve.

**Locality.** BAJA CALIFORNIA: Bahía de Los Ángeles^TL^: *Rhinobatos leucorhynchus*^TH^ (see Tyler 2001^OR^). BAJA CALIFORNIA SUR: Santa Rosalía: *Rhinobatos leucorhynchus* (see Tyler 2001).

**Specimens in collections.** CNHE (3920-22).

**Escherbothriidae Ruhnke, Caira & Cox, 2015**

***Escherbothrium molinae* Berman & Brooks, 1994**

**Site of infection.** Spiral valve.

**Locality.** GUERRERO: Bahía de Acapulco: *Urotrygon* sp. (see Zaragoza-Tapia et al. 2013).

**Specimens in collections.** CNHE (8513-4); HWML (49850-3).

**Eutetrarhynchidae Guiart, 1927**

***Dollfusiella litocephalus* (Heinz & Dailey, 1974) Beveridge, Neifar & Euzet, 2004**

**Site of infection.** Spiral valve.

**Locality.** BAJA CALIFORNIA: Bahía de San Quintín: *Triakis semifasciata* (see Heinz and Dailey 1974).

**Specimens in collections.** USNPC (072672).

**Notes.** The original denomination of this species was *Eutetrarhynchus litocephalus*, but it was transferred to the genus *Dollfusiella* by Beveridge et al. (2004).

***Dollfusiella cortezensis* (Friggens & Duszynski, 2005) Schaeffner, 2014**

**Site of infection.** Spiral valve.

**Locality.** SONORA: Puerto Peñasco^TL^: *Urolophus halleri*^TH^ (see Friggens and Duszynski 2005^OR^).

**Specimens in collections.** USNPC (92215).

**Notes. Notes.** Published as *Eutetrarhynchus* sp. in Friggens and Brown (2005). The original denomination of this species was *Eutetrarhynchus cortezensis*, but it was transferred to the genus *Dollfusiella* by Schaeffner (2014).

***Fellicocestus mobulae* Campbell & Beveridge, 2006**

**Site of infection.** Gall bladder.

**Locality.** BAJA CALIFORNIA SUR: Bahía de la Paz: *Mobula* sp. (see Campbell and Beveridge 2006a); Puntarena^TL^: *Mobula japanica*^TH^ (see Campbell and Beveridge 2006a^OR^).

**Specimens in collections.** CNHE (5452); USNPC (97899, 9700).

**Eutetrarhynchidae gen. sp.**

**Site of infection.** Spiral valve.

**Locality.** VERACRUZ: Playa de Chachalacas: *Carcharhinus leucas* (see Mendez and Dorantes 2013).

**Specimens in collections.** CNHE (6169).

***Hemionchos maior* Campbell & Beveridge, 2006**

**Site of infection.** Spiral valve.

**Locality.** BAJA CALIFORNIA SUR: Bahía de la Paz^TL^: *Mobula japanica*^TH^ (see Campbell and Beveridge 2006a^OR^).

**Specimens in collections.** CNHE (5466-7).

***Hemionchos mobulae* Campbell & Beveridge, 2006**

**Site of infection.** Spiral valve.

**Locality.** BAJA CALIFORNIA SUR: Bahía de la Paz^TL^, Puntarena: *Mobula japanica*^TH^ (see Campbell and Beveridge 2006a^OR^): Loreto, Santa Rosalía: *Mobula munkiana* (see Campbell and Beveridge 2006a).

**Specimens in collections.** CNHE (5465-6); LRP (3961); USNPC (97908-9).

***Hemionchos striatus* Campbell & Beveridge, 2006**

**Site of infection.** Spiral valve.

**Locality.** BAJA CALIFORNIA SUR: Bahía de la Paz^TL^: *Mobula japanica*, *Mobula thurstoni*^TH^ (see Campbell and Beveridge 2006a^OR^); Loreto: *Mobula thurstoni*, *Myliobatis californica* (see Campbell and Beveridge 2006a).

**Specimens in collections.** CNHE (5460); LRP (3948); USNPC (97904-6).

***Mecistobothrium myliobati* Heinz & Dailey, 1974**

**Site of infection.** Spiral valve.

**Locality.** SONORA: Puerto Peñasco: *Urolophus halleri* (see Friggens and Brown 2005).

**No specimens in collections.**

***Mobulocestus lepidoscolex* Campbell & Beveridge, 2006**

**Site of infection.** Nephridial system.

**Locality.** BAJA CALIFORNIA SUR: Bahía de la Paz^TL^: *Mobula thurstoni*^TH^ (see Campbell and Beveridge 2006a^OR^).

**Specimens in collections.** CNHE (5458); USNPC (97902).

***Mobulocestus mollis* Campbell & Beveridge, 2006**

**Site of infection.** Cloaca.

**Locality.** BAJA CALIFORNIA SUR: Bahía de la Paz^TL^: *Mobula thurstoni*^TH^ (see Campbell and Beveridge 2006a^OR^).

**Specimens in collections.** CNHE (5456).

***Mobulocestus nephriditis* Campbell & Beveridge, 2006**

**Site of infection.** Nephridial system.

**Locality.** BAJA CALIFORNIA SUR: Bahía de la Paz^TL^: *Mobula thurstoni*^TH^ (see Campbell and Beveridge 2006a^OR^).

**Specimens in collections.** CNHE (5454); USNPC (97901).

***Oncomegas paulinae* Toth, Campbell & Schmidt, 1992**

**Site of infection.** Spiral valve.

**Locality.** SONORA: Puerto Peñasco^TL^: *Urolophus halleri*^TH^ (see Toth et al. 1992^OR^).

**Specimens in collections.** BMNH (1991.10.30.1-3); USNPC (082186).

***Parachristianella dimegacantha* Krause, 1959**

**Site of infection.** Spiral valve.

**Locality.** BAJA CALIFORNIA SUR: Bahía de la Paz: *Dasyatis longa* (see Campbell and Beveridge 2007); Loreto: *Urotrygon simulatrix* (see Campbell and Beveridge 2007); San José del Cabo: *Sphyrna zygaena* (see Campbell and Beveridge 2007).

**Specimens in collections.** USNPC (97925-7).

***Parachristianella parva* Campbell & Beveridge, 2007**

**Site of infection.** Spiral valve.

**Locality.** BAJA CALIFORNIA SUR: Santa Rosalía^TL^: *Urolophus maculatus*^TH^ (see Campbell and Beveridge 2007^OR^).

**Specimens in collections.** CNHE (5472).

***Parachristianella trygoni* Dollfus, 1946**

**Site of infection.** Spiral valve.

**Locality.** BAJA CALIFORNIA: Bahía de Los Ángeles^TL^: *Dasyatis brevis*^TH^ (see Campbell and Beveridge 2007^OR^). BAJA CALIFORNIA SUR: Loreto: *Mobula munkiana* (see Campbell and Beveridge 2007).

**Specimens in collections.** USNPC (97923-4).

***Prochristianella minima* Hainz & Daily, 1974**

**Site of infection.** Spiral valve.

**Locality.** SONORA: Puerto Peñasco: *Urolophus halleri* (see Friggens and Brown 2005).

**Specimens in collections.** USNPC (92211, 92216).

***Prochristianella multidum* Friggens & Duzynski, 2005**

**Site of infection.** Spiral valve.

**Locality.** SONORA: Puerto Peñasco^TL^: *Urolophus halleri*^TH^ (see Friggens and Duszynski 2005^OR^).

**Specimens in collections.** USNPC (92218-9).

***Pseudochristianella elegantissima* Campbell & Beveridge, 2006**

**Site of infection.** Spiral valve.

**Locality.** BAJA CALIFORNIA: Puertecitos: *Dasyatis brevis* (see Campbell and Beveridge 2006b). BAJA CALIFORNIA SUR: Bahía de la Paz^TL^: *Dasyatis brevis*^TH^ (see Campbell and Beveridge 2006b^OR^); San José del Cabo: *Dasyatis longa* (see Campbell and Beveridge 2006b)

**Specimens in collections.** CNHE (5468); USNPC (97915-6).

***Pseudochristianella nudiscula* Campbell & Beveridge, 2006**

**Site of infection.** Spiral valve.

**Locality.** BAJA CALIFORNIA: Bahía de Los Ángeles: *Myliobatis longirostris*, *Rhinobatos productus* (see Campbell and Beveridge 2006b). BAJA CALIFORNIA SUR: Santa Rosalía^TL^: *Zapteryx exasperata*, *Rhinobatos productus*^TH^ (see Campbell and Beveridge 2006b^OR^); San José del Cabo: *Dasyatis longa* (see Campbell and Beveridge 2006b).

**Specimens in collections.** CNHE (5470); USNPC (97917, 97921-2).

**Lacistorhynchidae Guiart, 1927**

***Callitetrarhynchus gracilis* (Rudolphi, 1819)**

**Site of infection.** Spiral valve.

**Locality.** VERACRUZ: Playa Chachalacas: *Carcharhinus leucas* (see Méndez and Dorantes 2013).

**Specimens in collections.** CNHE (6867).

***Floriceps caballeroi* Cruz-Reyes, 1977**

**Site of infection.** Spiral valve.

**Locality.** SONORA: Laguna de Agiabampo^TL^: *Negaprion brevirostris*^TH^ (see Cruz-Reyes 1977^OR^).

**Specimens in collections.** CNHE (479-80).

**Notes.** According to Palm (2004), this material is a synonym of *Floriceps saccatus*. However, the poor condition of the type material re-examined during the present study precludes any conclusion about the taxonomic status of this species.

***Floriceps saccatus* Cuvier, 1817**

**Site of infection.** Spiral valve.

**Locality.** BAJA CALIFORNIA: NA: *Notorhynchus cepedianus* (see Heinz and Dailey 1974).

**No specimens in collections.**

**Litobothriidae Dailey, 1969**

***Litobothrium aenigmaticum* Caira, Jensen, Waeschenbach & Littlewood, 2014**

**Site of infection.** Spiral valve.

**Locality.** BAJA CALIFORNIA SUR: Santa María^TL^, Santa Rosalía: *Alopias pelagicus*^TH^ (see Caira et al. 2014a^OR^).

**Specimens in collections.** CNHE (8941-4).

***Litobothrium amplifica* (Kurochkin & Slankis, 1973) Euzet, 1994**

**Site of infection.** Spiral valve.

**Locality.** BAJA CALIFORNIA: Bahía de Los Ángeles: *Alopias pelagicus* (see Olson and Caira 2001). BAJA CALIFORNIA SUR: Santa Rosalía: *Alopias pelagicus* (see Olson and Caira 2001). OAXACA: Golfo de Tehuantepec^TL^: *Alopias superciliosus*^TH^ (see Kurochkin and Slankis 1973^OR^).

**Specimens in collections.** BMNH (2000.3.7.8.10); CNHE (4051); TINRO (72020).

**Notes.** This species was described as a member of the genus *Renyxa*, but Euzet (1994) considered *Renyxa* as a synonym of *Litobothrium*.

***Litobothrium daileyi* Kurochkin & Slankis, 1973**

**Site of infection.** Spiral valve.

**Locality.** BAJA CALIFORNIA: Bahía de Los Ángeles: *Alopias pelagicus* (see Olson and Caira 2001). BAJA CALIFORNIA SUR: Santa Rosalía: *Alopias pelagicus* (see Olson and Caira 2001). OAXACA: Golfo de Tehuantepec^TL^: *Alopias superciliosus*^TH^ (see Kurochkin and Slankis 1973^OR^).

**Specimens in collections.** CNHE (4050); TINRO (72012).

***Litobothrium janovyi* Olson & Caira, 2001**

**Site of infection.** Spiral valve.

**Locality.** BAJA CALIFORNIA SUR: Santa Rosalía^TL^: *Alopias superciliosus*^TH^ (see Olson and Caira 2001^OR^).

**Specimens in collections.** CNHE (4052-3).

***Litobothrium nickoli* Olson & Caira, 2001**

**Site of infection.** Spiral valve.

**Locality.** BAJA CALIFORNIA: Bahía de Los Ángeles^TL^: *Alopias pelagicus*^TH^ (see Olson and Caira 2001^OR^). BAJA CALIFORNIA SUR: Santa Rosalía: *Alopias pelagicus* (see Caira et al. 2014a).

**Specimens in collections.** CNHE (4054-55); LRP (8321).

**Lecanicephalidea*incertae sedis* (Family)**

***Aberrapex senticosus* Jensen, 2001**

**Site of infection.** Spiral valve.

**Locality.** BAJA CALIFORNIA SUR: Santa Rosalía^TL^: *Myliobatis californica*^TH^ (see Jensen 2001^OR^).

**Specimens in collections.** CNHE (4188); HWML (16374); USNPC (91208).

**Notes.** This species appears as *Discobothrium* n. sp. in Caira et al. (1999) and Caira et al. (2001).

***Healyum harenamica* Jensen, 2001**

**Site of infection.** Spiral valve.

**Locality.** BAJA CALIFORNIA SUR: Punta Arena^TL^: *Mobula japanica*^TH^ (see Jensen 2001^OR^).

**Specimens in collections.** CNHE (4186); HWML (16376); USNPC (91212).

***Healyum pulvis* Jensen, 2001**

**Site of infection.** Spiral valve.

**Locality.** BAJA CALIFORNIA SUR: Punta Arena^TL^: *Mobula japanica*^TH^ (see Jensen 2001^OR^).

**Specimens in collections.** CNHE (4184); HWML (16377); USNPC (91213).

**Notes.** This taxon appears as New genus 3 n. sp., in the phylogenetic analysis done by Caira et al. (2001).

***Paraberrapex manifestus* Jensen, 2001**

**Site of infection.** Spiral valve.

**Locality.** BAJA CALIFORNIA SUR: Santa Rosalía^TL^: *Squatina californica*^TH^ (see Jensen 2001^OR^).

**Specimens in collections.** CNHE (4179); HWML (16375); USNPC (91209).

**Notes.** *Paraberrapex manifestus* was referred to as New genus 2 n. sp. in the phylogenetic analysis done by Caira et al. (2001).

***Quadcuspibothrium francisi* Jensen, 2001**

**Site of infection.** Spiral valve.

**Locality.** BAJA CALIFORNIA SUR: Punta Arena^TL^: *Mobula japanica*^TH^ (see Jensen 2001^OR^).

**Specimens in collections.** CNHE (4182); HWML (16378); USNPC (91214).

**Notes.** This species was referred to as New genus 4 n. sp. in the phylogenetic analysis done by Caira et al. (2001).

**Tetragonocephalidae Yamaguti, 1959**

***Tylocephalum* sp.**

**Site of infection.** Spiral valve.

**Locality.** BAJA CALIFORNIA: Bahía de Los Ángeles: *Rhinoptera steindachneri* (see Caira et al. 1999). GUERRERO: Bahía de Acapulco: *Rhinoptera steindachneri* (see Carbajal-Violante 2012).

**Specimens in collections.** CNHE (8295-8296).

**Onchoproteocephalidea*incertae sedis* (Family)**

***Acanthobothrium bajaensis* Appy & Dailey, 1973**

**Site of infection.** Spiral valve.

**Locality.** BAJA CALIFORNIA: Bahía de San Quintín^TL^: *Heterodontus francisci*^TH^ (see Appy and Dailey 1973^OR^).

**Specimens in collections.** USNPC (72567-8).

***Acanthobothrium bullardi* Goshroy & Caira, 2001**

**Site of infection.** Spiral valve.

**Locality.** BAJA CALIFORNIA: Bahía de Los Ángeles^TL^, Puertecitos: *Dasyatis brevis*^TH^ (see Goshroy and Caira 2001^OR^). BAJA CALIFORNIA SUR: Santa Rosalía: *Dasyatis brevis* (see Goshroy and Caira 2001).

**Specimens in collections.** CNHE (4045-6); LRP (2060–2062); USNPC (90466-8).

***Acanthobothrium cleofanus* Monks, Brooks & Pérez-Ponce de León, 1996**

**Site of infection.** Spiral valve.

**Locality.** JALISCO: Bahía de Chamela^TL^: *Dasyatis longa*^TH^ (see Monks et al. 1996^OR^).

**Specimens in collections.** CNHE (2670-1); HWML; MNHG INV.

***Acanthobothrium dasi* Goshroy & Caira, 2001**

**Site of infection.** Spiral valve.

**Locality.** BAJA CALIFORNIA: Puertecitos^TL^: *Dasyatis brevis*^TH^ (see Goshroy and Caira 2001^OR^).

**Specimens in collections.** CNHE (4043-4); HWML (15549-51); LRP (2051-4); USNPC (90463-5).

***Acanthobothrium dollyae* Caira & Burge, 2001**

**Site of infection.** Spiral valve.

**Locality.** BAJA CALIFORNIA: Bahía de Los Ángeles^TL^, Isla San Esteban: *Diplobatis ommata*^TH^ (see Caira and Burge 2001^OR^). BAJA CALIFORNIA SUR: Punta Arena: *Diplobatis ommata* (see Caira and Burge 2001).

**Specimens in collections.** CNHE (4169-70); LRP (2097-2101); USNPC (90837-9).

***Acanthobothrium maryanskii* Caira & Burge, 2001**

**Site of infection.** Spiral valve.

**Locality.** BAJA CALIFORNIA SUR: Loreto^TL^, Punta Arena: *Diplobatis ommata*^TH^ (see Caira and Burge 2001^OR^).

**Specimens in collections.** CNHE (4171-2); LRP (2012-3); USNPC (90840-1).

***Acanthobothrium olseni* Dailey & Mudry, 1968**

**Site of infection.** Spiral valve.

**Locality.** Sonora: Puerto Peñasco: *Urolophus halleri* (see Friggens and Brown 2005).

**No specimens in collections.**

***Acanthobothrium parviuncinatum* Young, 1954**

**Site of infection.** Spiral valve.

**Locality.** BAJA CALIFORNIA: Puertecitos: *Urolophus halleri* (see Caira et al. 1999). SONORA: Puerto Peñasco: *Urolphus halleri* (see Friggens and Brown 2005).

**Specimens in collections.** CNHE (4171-2); LRP (2012-3); USNPC (90840-1).

***Acanthobothrium puertecitense* Caira & Zahner, 2001**

**Site of infection.** Spiral valve.

**Locality.** BAJA CALIFORNIA: Puertecitos: *Heterodontus francisci* (see Caira and Zahner 2001).

**Specimens in collections.** CNHE (4175-6); LRP (2105-6); USNPC (90843).

**Notes.** Caira et al. (2001) recorded this species as *Acanthobothrium* n. sp. 1.

***Acanthobothrium rajivi* Goshroy & Caira, 2001**

**Site of infection.** Spiral valve.

**Locality.** BAJA CALIFORNIA: Puertecitos^TL^: *Dasyatis brevis*^TH^ (see Goshroy and Caira 2001^OR^).

**Specimens in collections.** CNHE (4043-4); HWML (15552); LRP (2055-6); USNPC (90461).

***Acanthobothrium royi* Caira & Burge, 2001**

**Site of infection.** Spiral valve.

**Locality.** BAJA CALIFORNIA SUR: Loreto, Punta Arena^TL^: *Diplobatis ommata*^TH^ (see Caira and Burge 2001^OR^).

**Specimens in collections.** CNHE (4173-4); LRP (2014); USNPC (90842).

***Acanthobothrium santarosaliense* Caira & Zahner, 2001**

**Site of infection.** Spiral valve.

**Locality.** BAJA CALIFORNIA SUR: Santa Rosalía^TL^: *Heterodontus francisci*^TH^ (see Caira and Zahner 2001^OR^).

**Specimens in collections.** CNHE (4177-78); LRP (2107); USNPC (90844).

***Acanthobothrium soberoni* Goshroy & Caira, 2001**

**Site of infection.** Spiral valve.

**Locality.** BAJA CALIFORNIA: Bahía de Los Ángeles, Puertecitos^TL^: *Dasyatis brevis*^TH^ (see Goshroy and Caira 2001^OR^). BAJA CALIFORNIA SUR: Loreto: *Dasyatis brevis* (see Goshroy and Caira 2001).

**Specimens in collections.** CNHE (4040-1); HWML (15548); LRP (2057-9); USNPC (90462).

***Acanthobothrium* sp.**

**Site of infection.** Spiral valve.

**Locality.** BAJA CALIFORNIA: Bahía de Los Ángeles: *Urolophus halleri* (see Caira et al. 2001), Puertecitos: *Heterodontus francisci* (see Caira et al. 2001). BAJA CALIFORNIA SUR: Santa Rosalía: *Urolophus maculatus* (see Caira et al. 2001). NA: NA: *Dasyatis longa* (see Healy et al. 2009). SONORA: Puerto Peñasco: *Urolophus halleri* (see Friggens and Brown 2005).

**No specimens in collections.**

### No specimens in collections

***Acanthobothroides* sp.**

**Site of infection.** Spiral valve.

**Locality.** BAJA CALIFORNIA: Bahía de Los Ángeles: *Dasyatis brevis* (see Goshroy and Caira 2001). BAJA CALIFORNIA SUR: San José del Cabo: *Dasyatis brevis* (see Goshroy and Caira 2001).

**Specimens in collections.** CNHE (4048); USNPC (90439).

**Notes.** This material probably represents a new species as it differs from both *Acanthobothroides thorsoni* and *Acanthobothroides pacificus* (see Goshroy and Caira 2001).

***Onchobothrium* sp.**

**Site of infection.** Intestine.

**Locality.** BAJA CALIFORNIA: Ensenada: *Urolophus halleri* (HWML).

**Specimens in collections.** HWML (31324).

***Phoreibothrium* sp.**

**Site of infection.** Spiral valve.

**Locality.** BAJA CALIFORNIA: Bahía de Los Ángeles: *Carcharhinus leucas* (see Caira et al. 2001). NA: Golfo de México (Mexico): *Sphyrna mokarran* (see Caira et al. 2001). VERACRUZ: Playa Chachalacas: *Carcharhinus leucas* (see Mendez and Dorantes 2013).

**Specimens in collections.** CNHE (6866).

***Platybothrium angelbahiense* Healy, 2003**

**Site of infection.** Spiral valve.

**Locality.** BAJA CALIFORNIA: Bahía de Los Ángeles^TL^: *Carcharhinus leucas*^TH^ (see Healy 2003^OR^).

**Specimens in collections.** CNHE (4727-9); LRP (3213-3215); USNPC (92236).

***Platybothrium auriculatum* Yamaguti, 1952**

**Site of infection.** Intestine, spiral valve, stomach.

**Locality.** BAJA CALIFORNIA SUR: Bahía de La Paz, San Isidro: *Prionace glauca* (see Healy 2003); Las Barrancas, Punta Abreojos, Punta Belcher: *Prionace glauca* (see Méndez 2005).

**Specimens in collections.** CNHE (4730).

***Platybothrium* sp.**

**Site of infection.** Spiral valve.

**Locality.** BAJA CALIFORNIA: Bahía de Los Ángeles: *Carcharhinus leucas* (see Caira et al. 1999).

**Specimens in collections.** CSMNH.

**Notes.** Caira et al. (1999) identified this material as *Platybothrium cervinum*, but according to Healy (2003) these specimens represent an undescribed species.

***Platybothrium tantulum* Healy, 2003**

**Site of infection.** Spiral valve.

**Locality.** BAJA CALIFORNIA: Bahía de Los Ángeles: *Sphyrna lewini*, *Sphyrna cygaena* (see Healy 2003). BAJA CALIFORNIA SUR: San José del Cabo: *Sphyrna lewini* (see Healy 2003).

**Specimens in collections.** CNHE (4731-3).

***Prosobothrium armigerum* Cohn, 1902**

**Site of infection.** Intestine, stomach.

**Locality.** BAJA CALIFORNIA SUR: Punta Abreojos, Punta Belcher: *Prionace glauca* (see Méndez 2005).

**No specimens in collections.**

### No specimens in collections

**Otobothriidae Dollfus, 1942**

***Otobothrium* sp.**

**Site of infection.** Spiral valve.

**Locality.** BAJA CALIFORNIA SUR: San José del Cabo: *Sphyrna zygaena* (see Schaeffner and Beveridge 2013). VERACRUZ: Playa Chachalacas: *Carcharhinus leucas* (see Méndez and Dorantes 2005).

**Specimens in collections.** CNHE (6863-3).

**Phyllobothriidae Braun, 1900**

***Orygmatobothrium* sp.**

**Site of infection.** Spiral valve.

**Locality.** BAJA CALIFORNIA: Puertecitos: *Mustelus henlei* (see Caira et al. 1999).

**No specimens in collections.**

***Paraorygmatobothrium* sp.**

**Site of infection.** Spiral valve.

**Locality.** VERACRUZ: Playa Chachalacas: *Carcharhinus leucas* (see Méndez and Dorantes 2013).

**Specimens in collections.** CNHE (6864-5).

**Notes.** This material was recorded as *Paraorygmatobothrium* sp. 1 and sp. 2.

***Phyllobothrium hallericola* Church & Schmidt, 1990**

**Site of infection.** Spiral valve.

**Locality.** SONORA: Puerto Peñasco^TL^: *Urolophus halleri*^TH^ (see Church and Schmidt 1990^OR^).

**Specimens in collections.** USNPC (81051-2).

**Notes.** The accession number published by Church and Schmidt (1990) is wrong.

***Phyllobothrium* sp.**

**Site of infection.** Intestine, spiral valve, stomach.

**Locality.** BAJA CALIFORNIA SUR: Las Barrancas, Punta Abreojos, Punta Belcher: *Prionace glauca* (see Méndez 2005). GUERRERO: Bahía de Acapulco: *Rhinoptera steindachneri* (see Carbajal-Violante 2012). SONORA: Puerto Peñasco: *Urolophus halleri* (see Friggens and Brown 2005, Church and Schmidt 1990).

**Specimens in collections.** CNHE (8291-2); USNPC (81053).

**Notes.** The accession number published by Church and Schmidt (1990) is wrong.

**Pterobothriidae Pintner, 1931**

***Pterobothrioides carvajali* Campbell & Beveridge, 1997**

**Site of infection.** Spiral valve.

**Locality.** BAJA CALIFORNIA: Puertecitos^TL^: *Dasyatis longa*^TH^ (see Campbell and Beveridge 1997^OR^).

**Specimens in collections.** CNHE (3142); USNPC (85472).

**Rhinebothriidea*incertae sedis* (Family)**

***Serendip danbrooksi* Monks, Zaragoza-Tapia, Pulido-Flores & Violante-González, 2015**

**Site of infection.** Spiral valve.

**Locality.** GUERRERO: Bahía de Acapulco^TL^: *Rhinoptera steindachneri*^TH^ (Monks et al. 2015 ^OR^). SINALOA: Mazatlán: *Rhinoptera steindachneri* (Monks et al. 2015).

**Specimens in collections.** CNHE (8293-4; 9725-7); HWML (75091-104); MNGH-PLAT (90513-5).

**Notes.** This species appears as *Serendip* sp. in Carbajal-Violante (2012). According to Ruhnke et al. (2015) the genus *Serendip* is clearly a candidate for membership in the Rhinebothriidea; a molecular analysis will be necessary to assign it to family level.

**Rhinebothriidae Euzet, 1953**

***Glyphobothrium zwerneri* Williams & Campbell, 1977**

**Site of infection.** Spiral valve.

**Locality.** CAMPECHE: Ciudad del Carmen: *Rhinoptera bonasus* (see Pulido-Flores and Monks 2014).

**Specimens in collections.** CNHE (8838).

**Notes.** The inclusion of this cestode species in Rhinebothriidae follows Appeltans et al. (2012).

***Rhinebothrium chollaensis* Friggens & Duszynski, 2005**

**Site of infection.** Spiral valve.

**Locality.** SONORA: Puerto Peñasco (Bahía Cholla)^TL^: *Urolophus halleri*^TH^ (see Friggens and Duszynski 2005^OR^).

**Specimens in collections.** USNPC (92213-4).

**Notes.** Published as *Rhinebothrium* sp. in Friggens and Brown (2005).

***Rhinebothrium gravidum* Friggens & Duszynski, 2005**

**Site of infection.** Spiral valve.

**Locality.** SONORA: Puerto Peñasco^TL^: *Urolophus halleri*^TH^ (see Friggens and Duszynski 2005^OR^).

**Specimens in collections.** USNPC (92212).

**Notes.** Published as *Rhinebothrium* sp. in Friggens and Brown (2005).

***Rhinebothrium maccallumi* Linton, 1924**

**Site of infection.** Spiral valve.

**Locality.** NA: NA: *Dasyatis americana* (see Healy et al. 2009).

**No specimens in collections.**

**Notes.** Species identification requires verification according to Healy et al. (2009).

***Rhinebothrium* sp.**

**Site of infection.** Spiral valve.

**Locality.** BAJA CALIFORNIA: Puertecitos: *Dasyatis brevis* (see Healy et al. 2009). BAJA CALIFORNIA SUR: Loreto: *Urolophus maculatus* (see Caira et al. 1999); San José del Cabo: *Dasyatis longa* (see Healy et al. 2009).

**Specimens in collections.** LRP (3893, 3896).

**Notes.** The records of Healy et al. (2009) of *Dasyatis brevis* and *Dasyatis longa* were made as *Rhinebothrium* sp.5 and *Rhinebothrium* sp.6, respectively.

***Rhinebothrium urobatidium* (Young, 1955) Appy & Dailey, 1977**

**Site of infection.** Spiral valve.

**Locality.** SONORA: Puerto Peñasco: *Urolophus halleri* (see Friggens and Brown 2005).

**Specimens in collections.** USNPC (92202-5).

***Scalithrium* sp.**

**Site of infection.** Spiral valve.

**Locality.** BAJA CALIFORNIA SUR: San José del Cabo: *Dasyatis longa* (see Healy et al. 2009).

**Specimens in collections.** LRP (3895).

**Notes.** This record appears as *Scalithrium* n. sp. in Healy et al. (2009).

**Rhinoptericolidae Carvajal & Campbell, 1975**

***Rhinoptericola megacantha* Carvajal & Campbell, 1975**

**Site of infection.** Spiral valve.

**Locality.** GUERRERO: Bahía de Acapulco: *Rhinoptera steindachneri* (see Carbajal-Violante 2012).

**Specimens in collections.** CNHE (8297-8).

***Rhinoptericola* sp.**

**Site of infection.** Stomach.

**Locality.** GUERRERO: Bahía de Acapulco: *Rhinoptera steindachneri* (see Carbajal-Violante 2012).

**Specimens in collections.** CNHE (8299-8300).

### Tentaculariidae Poche, 1926

***Nybelinia anthicosum* Heinz & Dailey, 1974**

**Site of infection.** Spiral valve, stomach.

**Locality.** BAJA CALIFORNIA: Playa María: *Heterodontus francisci* (see Heinz and Dailey 1974). SONORA: Bahía de San Carlos: *Heterodontus francisci* (see Heinz and Dailey 1974).

**Specimens in collections.** USNPC (72675).

***Nybelinia* sp.**

**Site of infection.** Stomach.

**Locality.** BAJA CALIFORNIA SUR: Las Barrancas: *Prionace glauca* (see Méndez 2005).

**No specimens in collections.**

“**Tetraphyllidea” *incertae sedis* (Family)**

***Anthobothrium* sp.**

**Site of infection.** Intestine, stomach.

**Locality.** BAJA CALIFORNIA SUR: Punta Abreojos, Punta Belcher, Las Barrancas: *Prionace glauca* (see Méndez 2005).

**No specimens in collections.**

***Caulobothrium opisthorchis* Riser, 1955**

**Site of infection.** Spiral valve.

**Locality.** BAJA CALIFORNIA: Bahía de Los Ángeles: *Myliobatis californicus* (see Healy et al. 2009).

**Specimens in collections.** LRP (3910).

***Caulobothrium* sp.**

**Site of infection.** Spiral valve.

**Locality.** BAJA CALIFORNIA: Bahía de Los Ángeles: *Myliobatis californicus* (see Healy et al. 2009).

**Specimens in collections.** LRP (3012).

**Notes.** According to Healy et al. (2009) this material represents an undescribed species; recorded as *Caulobothrium* n. sp. 1 in Caira et al. (1999) and Caira et al. (2001).

***Duplicibothrium cairae* Ruhnke, Curran & Holbert, 2000**

**Site of infection.** Spiral valve.

**Locality.** BAJA CALIFORNIA: Bahía de los Ángeles, Puertecitos: *Rhinoptera steindachneri* (see Ruhnke et al. 2000). BAJA CALIFORNIA SUR: Santa Rosalía^TL^: *Rhinoptera steindachneri*^TH^ (see Ruhnke et al. 2000^OR^).

**Specimens in collections.** CNHE (3846-7); HWML (15275,15276); USNPC (89726-7).

**Notes.** This species was reported as *Duplicibothrium* n. sp. 1 in the phylogenetic analysis done by Olson et al. (1999).

***Duplicibothrium paulum* Ruhnke, Curran & Holbert, 2000**

**Site of infection.** Spiral valve.

**Locality.** BAJA CALIFORNIA: Bahía de los Ángeles, Puertecitos^TL^: *Rhinoptera steindachneri*^TH^ (see Ruhnke et al. 2000^OR^).

**Specimens in collections.** CNHE (3848); HWML (15277, 15278); USNPC (89728-9).

**Notes.** This species was reported as *Duplicibothrium* n. sp. 2 in the phylogenetic analysis done by Olson et al. (1999).

***Pedibothrium brevispine* Linton, 1909**

**Site of infection.** Spiral valve.

**Locality.** BAJA CALIFORNIA SUR: San José del Cabo: *Ginglymostoma cirratum* (see Caira and Euzet 2001).

**Specimens in collections.** CNHE (4191).

***Pedibothrium manteri* Caira, 1992**

**Site of infection.** Spiral valve.

**Locality.** BAJA CALIFORNIA SUR: San José del Cabo: *Ginglymostoma cirratum* (see Caira and Euzet 2001).

**Specimens in collections.** CNHE (4190).

***Symcallio evani* (Caira, 1985) Bernot, Caira & Pickering, 2015**

**Site of infection.** Spiral valve.

**Locality.** BAJA CALIFORNIA: Bahía de Los Ángeles: *Mustelus lunulatus* (see Nasin et al 1997); Puertecitos^TL^: *Mustelus lunulatus*^TH^ (see Caira 1985^OR^). BAJA CALIFORNIA SUR: San José del Cabo, Santa Rosalía: *Mustelus lunulatus* (see Nasin et al. 1997).

**Specimens in collections.** CNHE (3071); USNPC (78600, 87127).

**Notes.** This species was described as *Calliobothrium evani* and recently transferred to *Symcallio* by Bernot et al. (2015). Type host of *Symcallio evani* was determined by Caira (1985) as “unidentified shark of the family Carcharhinidae”; its accurate specific identity was established by Nasin et al. (1997).

***Symcallio riseri* (Nasin, Caira & Euzet, 1997) Bernot, Caira & Pickering, 2015**

**Site of infection.** Spiral valve.

**Locality.** BAJA CALIFORNIA: Puertecitos: *Mustelus henlei* (see Caira 1985, Nasin et al. 1997). BAJA CALIFORNIA SUR: Santa Rosalía^TL^: *Mustelus henlei*^TH^ (see Nasin et al. 1997^OR^).

**Specimens in collections.** CNHE (3068-70); HWML (22537).

**Notes.** Specimens from Puertecitos were identified by Caira (1985) as *Calliobothrium lintoni* Euzet, 1954 and re-identified by Nasin et al. (1997) as *Calliobothrium riseri*. This species was recently transferred to *Symcallio* by Bernot et al. (2015).

**Nematoda Cobb, 1932**

**Gnathostomatidae Lane, 1923**

***Echinocephalus jenzeni* Hoberg, Brooks, Molina & Erbe, 1998**

**Site of infection.** Spiral valve.

**Locality.** CHIAPAS: Laguna Mar Muerto: *Himantura pacifica* (see Hoberg et al. 1998).

**Specimens in collections.** CNHE (2642).

***Echinocephalus pseudouncinatus* Millemann, 1951**

**Site of infection.** Spiral valve.

**Locality.** BAJA CALIFORNIA: Bahía San Francisquito, Isla Ángel de la Guarda (Puerto Refugio): *Heterodontus francisci* (see Milleman 1963); Bahía San Felipe: *Myliobatis californica* (see Milleman 1963). BAJA CALIFORNIA SUR: Laguna Ojo de Liebre (Guerrero Negro), Punta Abreojos: *Heterodontus francisci* (see Gómez del Prado-Rosas 1984).

**Specimens in collections.** CNHE (2289); USNPC (57448, 57450-2).

**Annelida Lamarck 1809**

**Hirudinea Lamarck, 1818**

**Piscicolidae Johnston, 1865**

**Piscicolidae gen. sp.**

**Site of infection.** Skin.

**Locality.** BAJA CALIFORNIA: Isla San Esteban: *Zapteryx exasperata* (CNHE).

**Specimens in collections.** CNHE (4027).

**Notes.** This specimen was deposited at the CNHE by Steve Curran as the holotype of the new species *Pseudaustrobdella cairae*, but their description was not published.

***Stibarobdella macrothela* (Schmarda, 1861) Llewellyn, 1966**

**Site of infection.** Skin.

**Locality.** TAMAULIPAS: Matamoros: *Ginglymostoma cirratum* (CNHE). VERACRUZ: Isla de Sacrificios: Elasmobranquio no determinado (CNHE).

**Specimens in collections.** CNHE (1640, 5572).

### Host-parasite list

**Selachii**

ALOPIIDAE

***Alopias pelagicus* Nakamura**

*Litobothrium aenigmaticum* (C)

*Litobothrium amplifica* (C)

*Litobothrium daileyi* (C)

*Litobothrium nickoli* (C)

*Paronatrema vaginicola* (T)

***Alopias superciliosus*** Lowe

*Litobothrium amplifica* (C)

*Litobothrium daileyi* (C)

*Litobothrium janovyi* (C)

CARCHARHINIDAE

***Carcharhinus brachyurus*** Günther

*Liomos winteri* (M)

***Carcharhinus leucas*** (Müller & Henle)

*Cathetocephalus resendezi* (C)

*Cathetocephalus thatcheri* (C)

*Callitetrarhynchus gracilis* (C)

Eutetrarhynchidae gen. sp. (C)

*Phoreiobothrium* sp. (C)

*Platybothrium angelbahiense* (C)

*Platybothrium* sp. (C)

*Otobothrium* sp. (C)

*Paraorygmatobothrium* sp. (C)

***Carcharhinus limbatus*** Müller & Henle

*Staphylorchis pacifica* (T)

***Negaprion brevirostris*** (Poey)

*Floriceps caballeroi* (C)

***Prionace glauca*** (Linnaeus)

*Anthobothrium* sp. (C)

*Helicometrina nimia* (T)

*Nybelinia* sp. (C)

*Paronatrema vaginicola* (T)

*Phyllobothrium* sp. (C)

*Platybothrium auriculatum* (C)

*Prosobothrium armigerum* (C)

GINGLYMOSTOMATIDAE

***Ginglymostoma cirratum*** (Bonnaterre)

*Pedibothrium brevispine* (C)

*Pedibothrium manteri* (C)

*Stibarobdella macrothela* (H)

HETERODONTIDAE

***Heterodontus francisci*** (Girard)

*Acanthobothrium bajaensis* (C)

*Acanthobothrium puertecitense* (C)

*Acanthobothrium santarosaliense* (C)

*Acanthobothrium* sp. (C)

*Echinocephalus pseudouncinatus* (N)

*Nybelinia anthicosum* (C)

*Otodistomum veliporum* (T)

***Heterodontus mexicanus*** Taylor & Castro-Aguirre

*Otodistomum veliporum* (T)

HEXANCHIDAE

***Notorynchus cepedianus*** (Péron)

*Floriceps saccatus* (C)

SPHYRNIDAE

***Sphyrna lewini*** (Griffith & Smith)

*Loimosina parawilsoni* (M)

*Platybothrium tantulum* (C)

***Sphyrna mokarran*** (Rüppell)

*Phoreiobothrium* sp. (C)

***Sphyrna zygaena* (L.)**

*Otobothrium* sp. (C)

*Parachristianella dimegacantha* (C)

*Platybothrium tantulum* (C)

SQUATINIDAE

***Squatina californica*** Ayres

*Otodistomum veliporum* (T)

*Paraberrapex manifestus* (C)

TRIAKIDAE

***Galeorhinus galeus*** (Linnaeus)

*Staphylorchis pacifica* (T)

***Mustelus californicus*** Gill

*Calicotyle californiensis* (M)

*Ptychogonimus megastomum* (T)

***Mustelus henlei*** (Gill)

*Calliobothrium evani* (C)

*Calliobothrium riseri* (C)

*Orygmatobothrium* sp. (C)

*Otodistomum veliporum* (T)

***Mustelus lunulatus*** Jordan & Gilbert

*Calliobothrium evani* (C)

*Probolitrema richiardii* (T)

*Ptychogonimus megastomum* (T)

***Triakis semifasciata* Girard**

*Dollfusiella litocephalus* (C)

**Undetermined shark**

*Staphylorchis pacifica* (T)

Undetermined Elasmobranchii

*Staphylorchis pacifica* (T)

*Stibarobdella macrothela* (H)

**Batoidea**

ANACANTHOBATIDAE

***Anacanthobatis folirostris*** Bigelow & Schroeder

*Calicotyle kroyeri* (M)

DASYATIDAE

***Dasyatis americana*** Hildebrand & Schroeder

*Dendromonocotyle octodiscus* (M)

*Rhinebothrium maccallumi* (C)

***Dasyatis brevis*** (Garman)

*Acanthobothrium bullardi* (C)

*Acanthobothrium dasi* (C)

*Acanthobothrium rajivi* (C)

*Acanthobothrium soberoni* (C)

*Acanthobothroides* sp. (C)

*Anaporrhutum euzeti* (T)

*Anthocephalum currani* (C)

*Listrocephalos kearni* (M)

*Parachristianella trygonis* (C)

*Probolitrema richiardii* (T)

*Prosorhynchus truncatus* (T)

*Pseudochristianella elegantissima* (C)

*Rhinebothrium* sp. (C)

***Dasyatis dipterura* (Jordan & Gilbert)**

*Anthocephalum currani* (C)

***Dasyatis longa*** (Garman)

*Acanthobothrium cleofanus* (C)

*Acanthobothrium* sp. (C)

*Anaporrhutum euzeti* (T)

*Anthocephalum lukei* (C)

*Anthocephalum michaeli* (C)

*Dasyonchocotyle dasyatis* (M)

*Listrocephalos wittingtoni* (M)

*Parachristianella dimegacantha* (C)

*Probolitrema richiardii* (T)

*Pseudochristianella elegantissima* (C)

*Pseudochristianella nudiscula* (C)

*Pterobothrioides carvajali* (C)

*Rhinebothrium* sp. (C)

*Scalithrium* sp. (C)

***Himantura pacifica*** Beebe & Tee-Van

*Echinocephalus jenzeni* (N)

MYLIOBATIDAE

***Aetobatus narinari*** Euphrasen

*Decacotyle floridana* (M)

***Mobula japanica*** (Müller & Henle)

*Fellicocestus mobulae* (C)

*Healyum harenamica* (C)

*Healyum pulvis* (C)

*Hemionchos maior* (C)

*Hemionchos mobulae* (C)

*Hemionchos striatus* (C)

*Quadcuspibothrium francisi* (C)

*Syncoelium vermilionense* (T)

***Mobula munkiana*** Notarbartolo-di-Sciara

*Anaporrhutum euzeti* (T)

*Hemionchos mobulae* (C)

*Parachristianella trygonis* (C)

***Mobula* sp.**

*Fellicocestus mobulae* (C)

***Mobula thurstoni*** (Lloyd)

*Hemionchos striatus* (C)

*Mobulocestus lepidoscolex* (C)

*Mobulocestus mollis* (C)

*Mobulocestus nephriditis* (C)

*Nagmia cisloi* (T)

*Syncoelium vermilionense* (T)

***Myliobatis californica*** Gill

*Aberrapex senticosus* (C)

*Anaporrhutum euzeti* (T)

*Caulobothrium opisthorchis* (C)

*Caulobothrium* sp. (C)

*Echeneibothrium* sp. (C)

*Echinobothrium fautleyae* (C)

*Echinobothrium mexicanum* (C)

*Echinocephalus pseudouncinatus* (N)

*Hemionchos striatus* (C)

*Probolitrema richiardii* (T)

***Myliobatis longirostris*** Applegate & Fitch

*Anaporrhutum euzeti* (T)

*Echinobothrium mexicanum* (C)

*Probolitrema richiardii* (T)

*Pseudochristianella nudiscula* (C)

***Rhinoptera bonasus*** (Mitchill)

*Benedeniella posterocolpa* (M)

*Euzetia lamothei* (M)

*Glyphobothrium zwerneri* (C)

***Rhinoptera steindachneri*** Evermann & Jenkins

Dasybatotreminae gen. sp. (M)

*Denarycotyle gardneri* (M)

*Duplicibothrium cairae* (C)

*Duplicibothrium paulum* (C)

*Echinobothrium fautleyae* (C)

*Heterocotyle* sp. (M)

Monocotylidae gen. sp. (M)

*Phyllobothrium* sp. (C)

*Rhinoptericola megacantha* (C)

*Rhinoptericola* sp. (C)

*Serendip danbrooksi* (C)

*Tylocephalum* sp. (C)

NARCINIDAE

***Diplobatis ommata*** (Jordan & Gilbert)

*Acanthobothrium dollyae* (C)

*Acanthobothrium maryanskii* (C)

*Acanthobothrium royi* (C)

*Anaporrhutum euzeti* (T)

***Narcine entemedor*** Jordan & Starks

*Anaporrhutum euzeti* (T)

*Nagmia rodmani* (T)

RAJIDAE

***Dipturus olseni*** Bigelow & Schroeder

*Calicotyle kroyeri* (M)

***Raja velezi*** Chirichigno

*Echeneibothrium* sp. (C)

RHINOBATIDAE

***Rhinobatos glaucostigma*** Jordan & Gilbert

*Anoplocotyloides papillatus* (M)

*Spinuris mexicana* (M)

***Rhinobatos lentiginosus*** Garman

*Paramonilicaecum* gen. sp. (T)

***Rhinobatos leucorhynchus*** Günther

*Echinobothrium rayallemangi* (C)

*Probolitrema richiardii* (T)

***Rhinobatos productus*** Ayres

*Anaporrhutum euzeti* (T)

*Pseudochristianella nudiscula* (C)

*Spinuris lophosoma* (M)

***Zapteryx exasperata*** (Jordan & Gilbert)

*Anaporrhutum euzeti* (T)

Piscicolidae gen. sp. (H)

*Pseudochristianella nudiscula* (C)

*Spinuris zapterygis* (M)

UROTRYGONIDAE

***Urobatis concentricus*** Osburn & Nichols

*Listrocephalos guberleti* (M)

*Echinobothrium hoffmanorum* (C)

***Urobatis jamaicensis*** Cuvier

*Dendromonocotyle octodiscus* (M)

***Urobatis*** sp.

*Listrocephalos guberleti* (M)

*Probolitrema richiardii* (T)

***Urolophus halleri*** Cooper

*Acanthobothrium olseni* (C)

*Acanthobothrium parviuncinatum* (C)

*Acanthobothrium* sp. (C)

*Anaporrhutum euzeti* (T)

*Anthocephalum duszynskii* (C)

*Calicotyle urobati* (M)

*Dollfusiella cotezensis* (C)

*Echinobothrium hoffmanorum* (C)

*Listrocephalos guberleti* (M)

*Mecistobothrium myliobati* (C)

*Onchobothrium* sp. (C)

*Oncomegas paulinae* (C)

*Phyllobothrium hallericola* (C)

*Phyllobothrium* sp. (C)

*Prochristianella minima* (C)

*Prochristianella multidum* (C)

*Rhinebothrium chollaensis* (C)

*Rhinebothrium gravidum* (C)

*Rhinebothrium urobatidium* (C)

***Urolophus maculatus*** (Garman)

*Acanthobothrium* sp. (C)

*Anaporrhutum euzeti* (T)

*Anthocephalum michaeli* (C)

*Calicotyle urobati* (M)

*Echinobothrium hoffmanorum* (C)

*Listrocephalos guberleti* (M)

*Parachristianella parva* (C)

*Pleorchis magniporus* (T)

*Probolitrema richiardii* (T)

*Rhinebothrium* sp. (C)

***Urotrygon simulatrix* Miyake & Eachran**

*Parachristianella dimegacantha* (C)

***Urotrygon* sp.**

*Escherbothrium molinae* (C)

“Mantarraya gris”

*Dendromonocotyle cortesi* (M)

## Discussion

To date, 132 helminth taxa (110 named species and 22 taxa not assigned to species) have been reported as parasites of elasmobranch species in Mexico. Seventy-three of these taxa are represented by holotypes from Mexican waters. All of these taxa have been collected in the adult stage (132). Thus, the richness of helminth species parasitizing elasmobranchs distributed in Mexican waters represents 7.2% of the worldwide species richness for this group (see Caira et al. 2012).

The 132 taxa parasitize 48 taxa of elasmobranchs (4 of them not assigned to species), within 15 families; Myliobatidae (8 species) and Urotrygonidae (6) being the families with the highest number of species sampled, due to the fact that100% and 60% respectively of the species of these two families recorded in Mexico, have been studied for helminths. In addition, helminths have been reported from 9 of the 12 orders of elasmobranchs in Mexican waters; no records are available for Squaliformes, Orectolobiformes (Selachii) or Rhinobatiformes (Batoidea). Fifteen of the 23 families of sharks have not been reported as hosts of helminths, as well as half of the families of rays. From the 204 known species of elasmobranchs recorded in Mexican waters, only 26% of them have been studied for helminths; thus, only 18.3% and 32.6% of shark and ray species, respectively, have been examined for, and found to host, helminths (Table 1). This value is similar to that found by Randhawa and Poulin (2010), who established that only 317 species (26%) of this globally distributed group of hosts have been examined for intestinal parasites (specifically tapeworms).

The species of elasmobranchs with the higher parasite species richness are *Urolophus halleri* (with 19 taxa), *Dasyatis longa* (14) and *Dasyatis brevis* (13). However, 8 shark and 9 ray species have been recorded only once as hosting helminths. In total, Batoidea is parasitized by 109 taxa of helminths and Selachii by 52, of which 56% and 61%, respectively, are cestode species. The mean value of species harbored by a host is 2.8 for sharks and 3.8 for rays; these traits are in accordance with the findings reported by Randhawa and Poulin (2010), who noted that, on average, batoids harbor significantly more species of tapeworms than sharks.

*Anaporrhutum euzeti* and *Probolitrema richardii* (Trematoda) are the species with the broadest host spectrum; the former species is associated with 11 species of rays from three localities, and the latter has been found in 7 species of rays and one shark from three localities. Higher host specificity was shown by cestodes; 62 of the 76 nominal species of this group were specialists for a particular species of elasmobranch. These results are in accordance with Caira and Jensen (2014) who noted that the majority of tapeworm species are extremely host-specific, exhibiting species-specific (i.e., oioxenous) associations with their hosts. However, more conclusive results can be obtained only by increasing the sampling of this group of vertebrates on both coasts of Mexico, through comprehensive studies in which complete necropsies of elasmobranchs are conducted, avoiding partial analysis of a particular site of infection or organ system, which is a common trait of the research in this field according to Caira et al. (2012).

*Dendromonocotyle octodiscus* had the widest geographic distribution, being found in 7 localities; this monogenean is followed by *Echinobothrium fautleyae*, *Anthocephalum michaeli* and *Staphylorchis pacifica*, which are distributed in 5 localities each, as well as *Symcallio evani* and *Calicotyle urobati*, recorded in 4 locations each. *Acanthobothrium* is the most specious genus, as it is represented by 14 species parasitizing 6 species of elasmobranchs.

**Figure 2.**
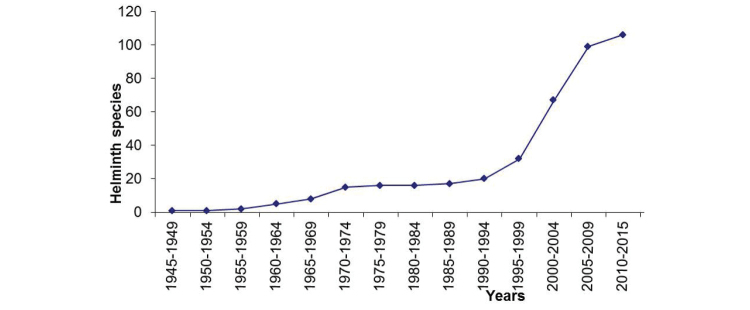
Cumulative curve of helminth species recorded in Mexico over 70 years of research.

Along with the increase in the number of species described worldwide, the number of helminth species parasitizing sharks and rays recorded in Mexico has increased in the past 2 decades, after slow growth from 1945, when Caballero y Caballero (1945) described the first species associated with this group of hosts (*Staphylorchis pacifica*). Between 1945 and 1994, only 20 species were reported in this group of hosts in the country. From 1995 to the present, this number increased more than 400%, rising to 107 species (Figure 2). According to Caira and Jensen (2014), approximately 250 species were erected over the past 2 decades; 36 of them were collected from elasmobranchs inhabiting Mexican waters.

The helminthological record of elasmobranchs distributed in Mexico is asymmetrically constituted in terms of the helminth groups represented, the hosts studied and the geographical distribution of the sampling sites. Cestodes are the most widely represented group, with 76 named species and 18 not assigned to species. The main reasons that explain this asymmetry can be summarized in two points: 1) the great diversity of cestodes associated with elasmobranchs, as nine of the 19 orders included in this Class infect this group of hosts, and eight are even exclusive parasites of them (Caira et al. 2014b); cestodes are by far the most diverse group of metazoan parasites of elasmobranchs, representing more than half of the described species for this host group (Caira et al. 2012); 2) the particular interest of a research team lead by Janine N. Caira from the University of Connecticut to inventory the fauna of tapeworm parasites of sharks and rays distributed in the Gulf of California, through the project “A systematic survey of the metazoan parasites of elasmobranchs from the Sea of Cortez” between 1993 and 1994. As a result of this project, more than 45 species of cestode were recorded in this area of Mexico, 36 of which were described as new species. The most intensively studied host group is Batoidea, with 32% of the species in the country harboring at least 1 species of helminth; on the other hand, only 18% of the species of sharks caught in Mexico have been reported as hosting helminths. To determine if this could represent a bias in sampling and not a reflection of the real richness of the helminths in the different groups of hosts, more sampling efforts are necessary. Likewise, the specific richness of helminths is concentrated in two states, i.e., Baja California Sur (69 helminth species reported to date) and Baja California (54), both located in the Gulf of California, up to now, the most intensively sampled region of Mexico.

In addition to the 132 helminth taxa recorded so far in elasmobranchs inhabiting Mexican waters, another 8 taxa of helminths were found in this group of hosts: 2 acanthocephalans, *Corynosoma* sp. (Méndez 2005) and *Gorgorhynchoides bullocki* (Monks et al. 2009), and 6 nematodes, *Anisakis simplex*, *Hedruris* sp. (Méndez 2005), *Anisakis* sp., *Hysterothylacium* sp., *Terranova* sp. (Pérez-Ponce de León et al. 1999), and *Mexiconema cichlasomae* (Moravec et al. 1998). However, their presence in elasmobranchs is considered accidental; elasmobranchs can be infected through 2 ways: 1) ingestion of prey acting as intermediate hosts for almost completely developed larvae and 2) ingestion of definitive hosts constituting an accidental, probably postcyclic transmission (Moravec et al. 1998; Anderson 2000; Weaver and Smales 2014).

In spite of the great amount of information generated in the last 20 years, new records of the helminth fauna of Elasmobranchii in Mexico remain scarce and fragmentary. To date, 81.7% of sharks and 67.4% of rays distributed in Mexican waters lack helminthological studies. Completing the helminthological inventory for this group of vertebrates is a major challenge, as recent estimates establish the number of species to be described associated with these hosts at approximately 3600, considering only the tapeworms (Randhawa and Poulin 2010). Only through efforts such as the one conducted by Caira and collaborators in the Gulf of California will a comprehensive understanding of these host-parasite associations be achieved.

**Table 2.** Sampled localities for elasmobranchs as hosts of helminths in Mexico.

**Baja California**	N	W
1) Bahía de Los Ángeles^†^	28°54'31"	113°29'47"
2) Bahía San Felipe	28°42'00"	112°35'00"
3) Bahía San Francisquito	29°45'05"	114°18'36"
4) Bahía de San Quintín	30°27'09"	115°56'54"
5) Ensenada	31°51'14"	116°37'45"
6) Isla Ángel de la Guarda (Puerto Refugio)	29°26'26"	113°34'25"
7) Isla Rasa	28°49'01"	112°58'25"
8) Isla San Esteban	28°41'39"	112°31'30"
9) Playa María	31°52'18"	116°39'31"
10) Puertecitos	30°20'59"	114°38'27"
**Baja California Sur**		
11) Bahía Almejas	24°31'00"	111°39'50"
12) Bahía de La Paz	24°14'30"	110°28'08"
13) Bahía de Santa Inés	27°02'55"	111°58'37"
14) Bahía Magdalena	25°20'00"	112°05'00"
15) Boca de Álamo	23°53'51"	109°48'12"
16) El Comitán	24°08'00"	110°25'00"
17) Isla Magdalena	24°15'00"	111°30'00"
18) Laguna Guerrero Negro (Ojo de Liebre)	27°51'21"	114°14'28"
19) Las Barrancas	26°00'30"	112°12'17"
20) Loreto	26°01'00"	111°19'50"
21) Punta Abreojos	26°27'45"	112°43'48"
22) Punta Arena	24°04'00"	109°50'00"
23) Punta Belcher	25°20'00"	112°05'00"
24) San Isidro	23°53'00"	109°47'00"
25) San José del Cabo	23°04'49"	109°40'49"
26) Santa María	27°24'53"	112°18'17"
27) Santa Rosalía	27°20'04"	112°15'35"
**Campeche**		
28) Bancos de Campeche	19°53'03"	90°31'43"
29) Ciudad del Carmen	19°51'33"	90°31'35"
30) Estuario Champotón	19°20'56"	90°41'18"
**Chiapas**		
31) Laguna Mar Muerto (El Paredón)	15°59'00"	94°00'00"
**Colima**		
32) Manzanillo	19°04'54"	104°19'31"
**Guerrero**		
33) Bahía de Acapulco	16°49'21"	99°52'55"
**Jalisco**		
34) Bahía de Chamela	19°33'15"	105°06'45"
35) Puerto Vallarta	20°35'48"	105°15'00"
**Nayarit**		
36) Punta Mita	20°46'38"	105°30'46"
37) San Blás	21°32'00"	105°17'22"
**Oaxaca**		
38) Golfo de Tehuantepec	15°45'26"	96°07'21"
**Quintana Roo**		
39) Blanquizal	18°16'03"	87°54'12"
40) Holbox	21°34'05"	86°14'32"
41) Isla Contoy	20° 48'25"	86° 47'15"
42) Isla Cozumel	20°24'10"	86°55'40"
43) Xcalak	18°19'32"	87°44'49"
**Sinaloa**		
44) Bahía Santa María	25°02'38"	108°05'14"
45) Mazatlán	23°14'03"	106°27'40"
**Sonora**		
46) Bahía de Guaymas	27°54'45"	110°52'41"
47) Bahía de San Carlos	27°56'36"	111°03'44"
48) Laguna de Agiabampo	26°21'54"	109°13'05"
49) Puerto Peñasco	31°18'33"	113°31'30"
50) Puerto Peñasco (Bahía Cholla)	31°20'00"	113°36'45"
**Tamaulipas**		
51) Matamoros	25°52'00"	97°30'00"
**Veracruz**		
52) Isla de Sacrificios	19°10'32"	96°05'50"
53) Playa Chachalacas	19°22'00"	96°22'00"
**Yucatán**		
54) Ría Lagartos	21°36'08"	88°08'51"

† These numbers correspond with the position of localities on Figure 1.
